# Iris Plateau Configuration With Secondary Pigment Dispersion: A Case Report

**DOI:** 10.7759/cureus.79107

**Published:** 2025-02-16

**Authors:** Jawaher Alwatban, Nouf A Zendi, Mofi M Walmany, Konrad Schargel

**Affiliations:** 1 Ophthalmology, King Khalid Eye Specialist Hospital and Research Center, Riyadh, SAU; 2 Ophthalmology, Dhahran Eye Specialist Hospital, Dammam, SAU

**Keywords:** angle closure glaucoma, glaucoma diagnosis and management, iris pigment dispersion, phacoemulsification cataract surgery, plateau iris, ultrasound biomicroscopy

## Abstract

Pigment dispersion (PD) and plateau iris configuration (PIC) are ocular conditions associated with increased intraocular pressure (IOP) and the development of glaucoma. PD involves the dispersion of pigment epithelium of the iris into the anterior chamber due to contact between the iris and zonular fibers, which subsequently blocks the trabecular meshwork and elevates the IOP. Anatomical variation in PIC narrows the anterior chamber angle. This case report aims to highlight a rare instance of concurrent PD secondary to PIC, conditions that are underreported despite their significant risk of glaucoma-related sequelae.

A 64-year-old male presented with blurred vision, pigmented cells in the anterior chamber, and optic disc cupping. Ultrasound biomicroscopy indicated a closed angle with 360-degree anterior rotation of the ciliary body, absence of ciliary sulcus, and chaffing of the posterior iris with the lens capsule and zonules. Collectively, these findings indicated PD secondary to PIC. The condition was managed with phacoemulsification and intraocular lens implantation to address the dual pathology and reduce the risk of glaucoma.

The co-occurrenceof PD and PIC necessitates vigilant monitoring and tailored therapeutic approaches to prevent irreversible optic nerve damage and vision loss. Additional studies are necessary to clarify the underlying mechanism and optimize diagnostic and treatment strategies for these complex ocular conditions. Early detection and collaborative management are essential in decreasing the risk of glaucoma and preserving visual function in affected individuals.

## Introduction

Plateau iris configuration (PIC) is characterized by a flat iris surface with a narrow angle and a relatively normal central anterior chamber depth [1]. During indentation gonioscopy, a "double hump sign" is observed [1]. PIC is defined on ultrasound biomicroscopy (UBM) by the anteriorly directed ciliary process, the steep rise and downward angulation of the iris root, the absence of a ciliary sulcus, and irido-angle contact in at least two quadrants [1-2]. Pigment dispersion (PD) refers to the release of iris pigment granules into the aqueous humor, which can occur due to mechanical friction between the posterior iris surface and anterior lens zonules or other intraocular structures [3]. This dispersion may be triggered by anatomical variations such as a concave iris configuration, increased iridolenticular contact, or excessive posterior bowing of the iris [3]. Other contributing factors include strenuous physical activity changes in pupillary dynamics, whether due to pupil dilation, or accommodation [4-5]. Additionally, genetic predisposition may influence the iris release of PD [6]. While often asymptomatic, excessive pigment liberation can lead to secondary complications, including increased trabecular meshwork pigmentation and intraocular pressure (IOP) fluctuations [7]. Early detection and treatment of PIC with secondary PD are critical to prevent irreversible damage to the optic nerve and loss of vision.

## Case presentation

**Figure 1:**
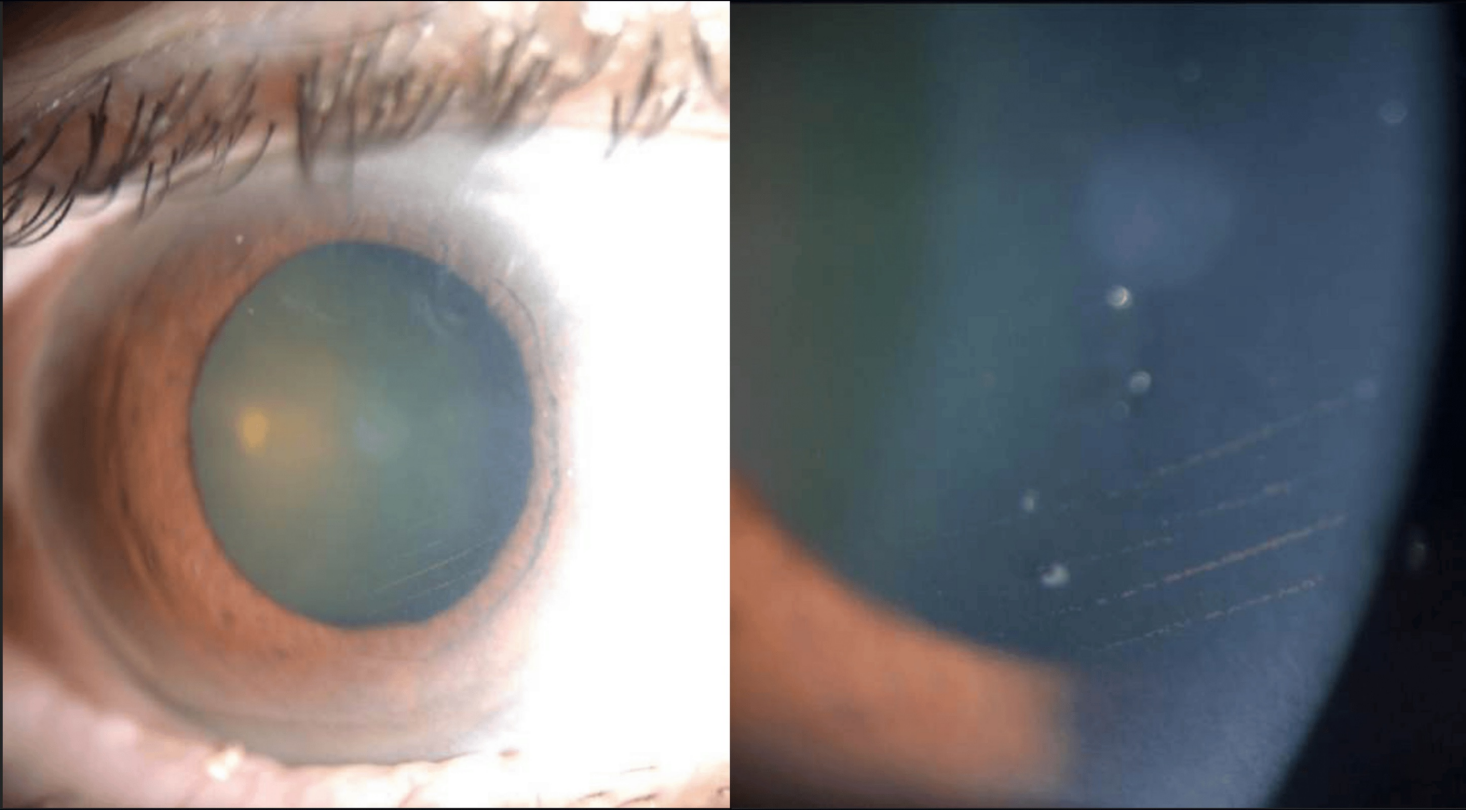
Slit lamp photographs of the right eye's cornea depicting horizontal pigmented lines within the endothelium.

A 64-year-old Middle Eastern male with type 2 diabetes mellitus (DM) presented to the glaucoma clinic with a complaint of blurry vision in both eyes. Two years before presenting to our clinic, the patient underwent complicated cataract surgery and pars plana vitrectomy for rhegmatogenous retinal detachment in the left eye, resulting in aphakia. Additionally, the patient had prior pan-retinal photocoagulation (PRP) marks in the left eye. There was no reported family history of ocular disease, use of medication (including antiglaucoma drops), or history of illicit drug use. Upon examination, his uncorrected visual acuity (VA) was 20/50 in the right eye and 4/200 in the left eye. With correction (+13.00-2.00x180), the left eye improved to 20/70. Goldman application tonometry indicated that IOP was 10 mmHg in the right eye and 15 mmHg in the left eye taken during a morning clinic visit. The central corneal thickness was 537 μm in the right eye and 556 μm in the left eye, as measured by pachymetry. Anterior segment examination of the right eye revealed a faint central scar and horizontal endothelial pigmented lines observed after performing indentation gonioscopy (Figure 1), which was absent in the left eye.

**Figure 2:**
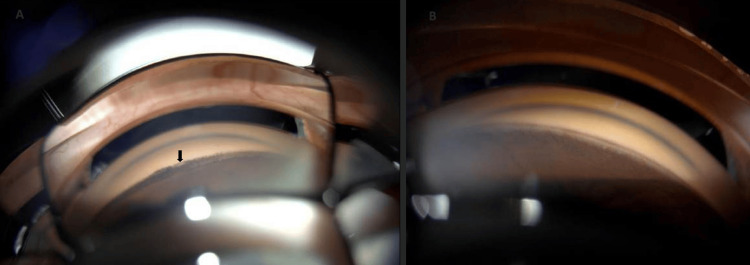
Pre-operative and post-operative gonioscopy views of the anterior chamber angle. (A) The pre-operative view shows a heavily pigmented angle. (B) The post-operative view shows an open angle with a notable decrease in pigment deposition across the angle.

The anterior chamber was deep centrally and shallow peripherally with +4 pigmented cells noted in the right eye prior to pupil dilation. The anterior chamber was deep and quiet in the left eye. The right eye exhibited nuclear sclerosis and posterior subcapsular cataracts, whereas the left eye was aphakic. Fundus examination revealed a myelinated retinal nerve fiber layer (MNFL) in the right eye with a cup-to-disc (C/D) ratio of 0.7 and mild non-proliferative diabetic retinopathy. The left eye had a C/D ratio of 0.65 with involuted proliferative diabetic retinopathy and evidence of full PRP. Indentation gonioscopy showed a double hump sign, with appositional-angle closure with heavily pigmented trabecular meshwork in the right eye (Figure 2). In the left eye, peripheral anterior synechia and angle closure were noted.

**Figure 3:**
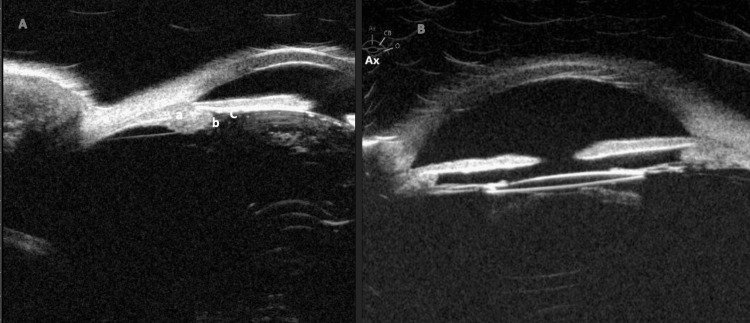
(A) Pre-operative UBM of the right eye showed a narrow angle with 360-degree anterior rotation of the ciliary body (a), absence of ciliary sulcus (b), and iridolenticular contact (c). (B) Post-operative UBM shows a notable deepening of the anterior chamber with loss of iridolenticular contact, while the ciliary body remains rotated and the anterior chamber angle is now open. UBM, ultrasound biomicroscopy

Humphrey’s visual field testing revealed an enlarged blind spot in the right eye, corresponding to the myelinated MNFL. In the left eye, superior and early inferior arcuate changes were observed, attributed to full PRP marks rather than glaucomatous damage. UBM of the right eye showed a closed angle with 360-degree anterior rotation of the ciliary body, absence of ciliary sulcus, and iridolenticular contact (Figure 3). In the aphakic left eye, UBM showed a similar 360-degree anterior rotation of the ciliary body. Both eyes had an axial length of 23 mm. These findings confirmed the diagnosis of iris plateau configuration leading to secondary PD.

The patient was advised to undergo cataract surgery and subsequently underwent phacoemulsification with intraocular lens implantation in the right eye. At the three-week postoperative follow-up, the visual acuity in the right eye improved to 20/25, with intraoperative pressures of 15 mmHg in the right eye and 17 mmHg in the left eye. A gonioscopy performed at the one-month postoperative visit showed an open angle and a significant reduction in pigment deposition within the angle (Figure 2).

## Discussion

To the best of our knowledge, no other cases have been published that document PIC with secondary PD. The prevalence of PIC in primary angle closure glaucoma is around 31% to 60%, and in open-angle glaucoma, it is approximately 19%; pigment dispersion syndrome (PDS) affects around 1-2% of the population [1,7]. In our scenario, the patient showed visually impairing cataracts while having PIC with secondary PD. PIC may contribute to PD through several hypothesized mechanisms. First, mechanical contact between the peripheral iris and zonules or lens can result in repetitive friction, particularly during the dynamic movement of the iris during accommodation, leading to pigment release from the posterior iris surface [3]. Second, intermittent angle closure, a characteristic feature of PIC, may further exacerbate friction and pigment shedding during episodes of angle closure [8]. Lastly, repetitive friction between the lens, zonules, and iris can cause minor ischemia or atrophy, increasing the iris’s susceptibility to pigment release under mechanical or pressure-induced stress. Together, these mechanisms provide a possible explanation of the link between PIC and PD.

The presence of Krukenberg’s spindles is a distinguishing feature of PDS [9]. According to Zeppieri et al., Krukenberg’s spindles are not pathognomonic and cannot be used as an indicative sign of PDS [7]. The vertical pattern of iris pigment deposits in the corneal endothelium (Krukenberg’s spindles) is caused by the convection currents of aqueous humor in the eye [10]. However, we observed horizontal pigment deposition only after indentation gonioscopy. The horizontal depositions are possibly due to altered aqueous flow dynamics caused by mechanical pressure disrupting the vertical convection currents. Additionally, indentation gonioscopy may have caused a transient rise in IOP, leading to reverse flow or stagnation of aqueous humor, contributing to the anomalous horizontal distribution of pigment on the endothelium. PIC is identified on UBM by anteriorly positioned ciliary processes, the absence of a ciliary sulcus, steep iris root angulation from its point of insertion, and iridotrabecular contact [1,2]. These features contribute to angle narrowing and are crucial for diagnosing PIC and differentiating it from other angle-closure mechanisms. The potential link between plateau iris and PD is intriguing but underreported due to several possible factors. Plateau iris is primarily identified through dynamic gonioscopy or imaging, techniques that may not always be performed unless specifically indicated. Additionally, PD can be episodic, with fluctuations in pigment release leading to inconsistent findings. In some cases, the presence of coexisting anatomical variations, such as a deep anterior chamber, may divert attention from subtle signs of PIC. Finally, early-stage or mild cases may not exhibit obvious clinical features, making detection even more challenging. A thorough examination is crucial for accurate diagnosis of PD. Numerous ocular conditions can mimic PD, including intraocular tumors and anterior uveitis [11-13]. Although anterior uveitis can present with symptoms similar to those of pigment dispersion (PD), such as pigment deposition and elevated IOP, the presence of inflammatory cells, a flare-up, redness, and photophobia can help differentiate anterior uveitis from PDS [11]. PD can indicate intraocular tumors, particularly those that affect the iris. However, a careful examination and imaging such as ultrasound or optical coherence tomography can aid in differentiation [12].

The patient was managed by phacoemulsification and intraocular lens implantation due to the absence of elevated IOP and to avoid the potential risk of increased inflammation associated with peripheral iridotomy. This approach aimed to reduce IOP, minimize PD, and improve visual acuity. An alternative option could include the combined therapeutic strategy of phacoemulsification and endoscopic cyclophotocoagulation (ECP), which further lowers IOP by reducing the size of the ciliary processes and creating space for the thickened peripheral iris during pupillary dilation. While phacoemulsification alone may decrease IOP, adding ECP enhances its efficacy [14-15].

However, the limitations of this report include the single-case nature and the lack of long-term follow-up data.

## Conclusions

In summary, we reported a unique case of a patient presenting with PIC with secondary PD. The lack of data on the prevalence of PD in individuals with PIC highlights the importance of our case. For management techniques to be optimized, clinical indicators must be precisely evaluated, and mimicking conditions must be accurately distinguished. Our case demonstrates the effective care of PD and PIC with phacoemulsification with intraocular lens implantation. The significant outcome of this combination strategy highlights its promise as a suitable therapeutic option for patients with concurrent PD and PIC.
